# Biomarkers of cardiometabolic complications in survivors of childhood acute lymphoblastic leukemia

**DOI:** 10.1038/s41598-020-78493-x

**Published:** 2020-12-09

**Authors:** Sophia Morel, Pauline Léveillé, Mariia Samoilenko, Anita Franco, Jade England, Nicolas Malaquin, Véronique Tu, Guillaume B. Cardin, Simon Drouin, Francis Rodier, Sarah Lippé, Maja Krajinovic, Caroline Laverdière, Daniel Sinnett, Geneviève Lefebvre, Emile Levy, Valérie Marcil

**Affiliations:** 1 Research Centre of Sainte-Justine University Health Center, Université de Montréal, 3175 Côte Ste-Catherine room 4.17.006, Montreal, QC H3T 1C5 Canada; 2 Department of Psychology, Université de Montréal, Montreal, QC H3T 1C5 Canada; 3 Department of Mathematics, Université du Québec à Montréal, Montreal, QC H3C 3P8 Canada; 4 Department of Pediatrics, Université de Montréal, Montreal, QC H3T 1C5 Canada; 5 Department of Nutrition, Université de Montréal, Montreal, QC H3T 1C5 Canada; 6 CRCHUM and Institut du Cancer de Montréal, Montreal, QC H2X 0A9 Canada; 7 Department of Radiology, Radio-Oncology, and Nuclear Medicine, Université de Montréal, Montreal, QC H3T 1C5 Canada

**Keywords:** Chemokines, Obesity, Metabolic syndrome, Predictive markers, Paediatric cancer

## Abstract

Survivors of childhood acute lymphoblastic leukemia (cALL) are at higher risk of developing cardiometabolic complications. We aimed at exploring the associations between biomarkers of inflammation, oxidative stress, endothelial function, endotoxemia and cardiometabolic risk factors. We conducted a cross-sectional analysis in 246 cALL survivors (mean age, 22.1 ± 6.3 years; mean time since diagnosis, 15.5 ± 5.2 years) and evaluated the associations using a series of logistic regressions. Using structural equation models, we also tested if the relationship between endotoxemia and cardiometabolic complications was mediated by the latent (unobserved) variable inflammation inferred from the observed biomarkers CRP, TNF-α and IL-6. High leptin-adiponectin ratio was associated with obesity [adjusted OR = 15.7; 95% CI (6.2–39.7)], insulin resistance [20.6 (5.2–82.1)] and the metabolic syndrome [11.2 (2.6–48.7)]. Higher levels of plasminogen activator inhibitor-1 and tumor necrosis factor-α were associated with obesity [3.37 (1.6–7.1) and 2.34 (1.3–4.2), respectively] whereas high C-reactive protein levels were associated with insulin resistance [3.3 (1.6–6.8)], dyslipidemia [2.6 (1.4–4.9)] and MetS [6.5 (2.4–17.9)]. Our analyses provided evidence for a directional relationship between lipopolysaccharide binding protein, related to metabolic endotoxemia, inflammation and cardiometabolic outcomes. Identification of biomarkers and biological mechanisms could open new avenues for prevention strategies to minimize the long-term sequelae, improve follow-up and optimize the quality of life of this high-risk population.

## Introduction

Survivors of childhood acute lymphoblastic leukemia (cALL) are at increased risk of long-term cardiometabolic complications including obesity, insulin resistance, dyslipidemia and hypertension^1–4^. While the precise etiology of these long-term complications is not fully understood, cranial radiation therapy (CRT) and chemotherapy have been proposed as contributing factors^3,5^. Possible underlying mechanisms include oxidative stress, chronic inflammation, adipose tissue dysfunction, endocrine disorders and accelerated cellular aging (reviewed in^6^). Identifying biomarkers related to long-term treatment outcomes could improve our knowledge on the biological factors influencing them and help predicting these morbidities in individuals and/or subgroups of patients.

In young adult survivors of cALL, the presence of systemic inflammation and increased activation of the immune system have been demonstrated^7–9^. In parallel, studies found high levels of adipokines and pro-inflammatory cytokines during^10–13^ and after^14,15^ chemotherapy, up to 5 years after the initiation of treatment^16^. Exposure to CRT can affect several metabolic pathways and thus promoting weight gain, insulin resistance and hormonal deficiencies^17^. Excess adipose tissue accompanying obesity promotes the synthesis and release of adipokines along with the development of a systemic inflammatory state, which may affect insulin sensitivity and vascular function^18^. Consequently, some adipokines including leptin and adiponectin have been proposed as biomarkers of metabolic disturbances in populations of childhood cancer survivors^19–22^, but all studies stressed the need for validating these findings in additional cohorts.

Endothelial dysfunction was reported in long-term survivors of cALL^23^, which seemingly contributes to their greater risk for cardiovascular disease development^24–28^. The infiltration and retention of low-density lipoprotein (LDL) in the arterial intima initiate an inflammatory endothelial process leading to atherosclerotic plaque formation. Modification of LDL through oxidation causes endothelial cells to express leucocyte adhesion molecules. In cALL survivors, circulatory levels of the adhesion molecules intracellular adhesion molecule 1 (ICAM-1)^29^, vascular adhesion molecule-1 (VCAM-1)^30^ and E-selectin^31^ were found elevated, although their relationship with cardiometabolic outcomes was not assessed.

Many chemotherapeutic agents induce oxidative stress and trigger an inflammatory response^7–9,32–34^. Oxidative stress is caused by the imbalance between the generation of free radicals and antioxidant defenses. If reactive oxygen species are not scavenged, they may conduct to widespread lipid, protein and DNA damage. Systemic inflammation also increases the susceptibility for oxidative modifications by endothelial and smooth muscle cells to LDL. To control the flux of reactive oxygen species, aerobic cells possess endogenous antioxidant enzymes that include superoxide dismutases (SOD) and glutathione peroxidases (GPx). Although several studies reported changes in antioxidant enzyme activities in serum leukocytes and red blood cells of leukemia patients during pathogenesis of leukemia and/or treatment (reviewed in^35^), findings are discordant and studies have not addressed these aspects in cancer survivors. Besides, mitochondrion is implicated in a variety of cellular functions including cell signaling, metabolism, cell death, aging, and cancer^36^. Variations in mitochondrial DNA (mtDNA)^37,38^ and in the regulation of mitochondrial proteins related to inflammation and to antioxidant activity have been identified in cALL survivors^39^, but no prior study has examined the relationship between mtDNA and cardiometabolic complications in this particular group of patients.

Endotoxins derived from intestinal bacteria are among the factors triggering a peripheral inflammatory state. Accordingly, the gut microbiota is recognized as a contributor to the systemic inflammation^40^ and oxidative stress^41^, which can remotely affect peripheral organs implicated in the development of obesity, insulin resistance and atherosclerosis^42,43^. Release of bacterial lipopolysaccharides (LPS), produced by Gram-negative bacteria, contribute to chronic^44^ and adipose tissue^45^ inflammation. LPS-binding protein (LBP) is produced mainly by the liver and helps mediate the LPS-induced inflammatory response^46^. Not only gut microbiota influence blood levels of LPS, but intestinal counts of Gram-negative bacteria was found correlated to LBP^47^. It is possible that endotoxemia, caused by changes in the intestinal microbiota during cALL treatment, triggers a pro-oxidative and pro-inflammatory state leading to the development of cardiometabolic complications.

There is a need to validate the utility of biomarker testing in order to characterize subgroups of patients most susceptible of developing late cardiometabolic complications. Therefore, our first objective was to explore the associations between blood biomarkers of oxidative stress, inflammation, endothelial function and endotoxemia and cardiometabolic risk factors in cALL survivors. Our second objective was to test, using structural equation models, if the relationship between endotoxemia and cardiometabolic complications was mediated by the latent (unobserved) variable inflammation inferred from the observed biomarkers CRP, TNF-α and IL-6.

## Results

### Cohort characteristics

Relevant demographic and treatment characteristics are outlined in Table 1. Median age at interview was 21.8 years, ranging from 8.5 to 41.0 years, and median time elapsed since diagnosis was 15.2 years. A total of 146 participants (59.4%) had received CRT (dose range: 10–19.8 Gy, with 47% of survivors having received ≥ 18 Gy). Dyslipidemia was the most prevalent cardiometabolic complication (41.5%), followed by obesity (32.5%), insulin resistance (17.1%) and pre-hypertention (pre-HTN) or hypertension (HTN) (12.2%). Among the types of anomalies that define dyslipidemia, 12.2% had elevated triglycerides, 17.5% high LDL-cholesterol (LDL-C) and 23.2% low high-density lipoprotein-cholesterol (HDL-C) levels. Additionally, metabolic syndrome (MetS) affected a total of 22 participants (9.0%).

**Table 1:** Demographic and clinical characteristics of participants.

	Total (n = 246)
**Sex N (%)**	
Male	121 (49.2)
Female	125 (50.8)
**Age at interview, years**	
Mean (SD)	22.1 (6.3)
Median (range)	21.8 (8.5–41.0)
**Age at cancer diagnosis, years**	
Mean (SD)	6.6 (4.6)
Median (range)	4.8 (0.9–18.0)
**Time since diagnosis, years**	
Mean (SD)	15.5 (5.2)
Median (range)	15.2 (5.4–28.2)
CRT exposure N (%)	146 (59.4)
**Cardiometabolic complication N (%)**	
Obesity	80 (32.5)
Insulin resistance	42 (17.1)
Pre–HTN/HTN	30 (12.2)
Dyslipidemia	102 (41.5)
Low HDL-C	57 (23.2)
High LDL-C	43 (17.5)
High TG	30 (12.2)
Metabolic syndrome	22 (9.0)

Metabolic syndrome was defined according to the International Diabetes Federation.

CRT cranial radiotherapy; HDL-C high-density lipoprotein-cholesterol; HTN hypertension; LDL-C low-density lipoprotein-cholesterol; SD standard deviation; TG triglycerides.

### Associations between biomarkers and cardiometabolic complications

In preliminary analyses, all biomarkers were tested in one third of participants and associations with cardiometabolic outcomes were assessed. Testing was carried out in additional participants when associations were significant or close to significance. Depending on the biomarkers, data of 79 to 244 participants were analyzed (Table 2).

**Table 2:** Median and range values of each blood biomarkers according to their functional group.

Biomarker	Median	Range
**Biomarkers of inflammation**		
Adiponectin (ng/ml) n = 176	14.8	1.02–43.9
Leptin (μg/ml) n = 157	11.3	0.43–50.0
Resistin (μg/ml) n = 87	4.88	1.03–13.3
Visfatin (pg/ml) n = 161	81.7	2.39–539
IL-6 (pg/ml) n = 237	0.36	0.01–9.01
TNF-α (pg/ml) n = 244	2.06	0.83–55.2
PAI-1 (ng/ml) n = 145	17.9	0.54–155
CRP (mg/l) n = 238	1.00	0.00–22.0
**Biomarkers of oxidative stress**		
SOD (U/ml) n = 156	105	55.8–167
GPx (nmol/min/ml) n = 244	632	139–1414
GSH (nmol/mg proteins) n = 240	42.3	18.6–77.1
Ox-LDL (U/l) n = 169	53.2	19.3–101
Protein carbonyls (nmol/mg) n = 87	0.59	0.11–1.90
mtDNA (relative gene expression) n = 227	4.68	2.15–7.56
**Biomarkers of endotoxemia**		
LPS (ng/ml) n = 125	12.5	2.20–26.6
LBP (μg/ml) n = 244	18.8	4.25–48.3
**Biomarkers of endothelial function**		
ICAM-1 (ng/ml) n = 244	537	284–1343
VCAM-1 (ng/ml) n = 244	695	365–1573
E-Selectin (ng/ml) n = 79	17.0	2.50–43.7

CRP: C-reactive protein; GPx glutathione peroxidase; GSH: glutathione; ICAM-1: intercellular adhesion molecule-1; mtDNA: mitochondrial DNA; LBP: lipopolysaccharide-binding protein; LPS: lipopolysaccharide; Ox-LDL: oxidized-low-density lipoprotein; PAI-1: plasminogen activator inhibitor-1; SOD: superoxide dismutase; TNF-α: tumor necrosis factor-α; VCAM-1: vascular cell adhesion molecule-1.

The associations between biomarkers of inflammation and cardiometabolic complications are outlined in Table 3. Adjusted models revealed that higher adiponectin levels were protective of obesity (OR = 0.18; 95% CI 0.09–0.38), insulin resistance (OR = 0.17; 95% CI 0.07–0.45) and dyslipidemia (OR = 0.34; 95% CI 0.18–0.66). Furthermore, participants with higher adiponectin levels were at lower risk of having MetS (OR = 0.07; 95% CI 0.01–0.38). Conversely, the risk of obesity and insulin resistance was associated with greater leptin levels (OR = 9.57; 95% CI 3.41–26.86 and OR = 13.17; 95% CI 3.68–47.10, respectively). The previous results are reflected in leptin-adiponectin ratio deleterious associations. The latter was associated with the risk of obesity (OR = 15.69; 95% CI 6.20–39.67), insulin resistance (OR = 20.60; 95% CI 5.17–82.14) and MetS (OR = 11.20; 95% CI 2.58–48.69). High PAI-1 levels were associated with the risk of having obesity (OR = 3.37; 95% CI 1.61–7.05). Higher CRP levels (> 3 mg/L vs. ≤ 3 mg/L) were associated with insulin resistance (OR = 3.27; 95% CI 1.58–6.79), dyslipidemia (OR = 2.61; 95% CI 1.40–4.86) and MetS (OR = 6.52; 95% CI 2.28–17.85). Participants with higher TNF-α levels were at increased risk of having obesity (OR = 2.34; 95% CI 1.31–4.18). Higher visfatin levels were protective of insulin resistance (OR = 0.27; 95% CI 0.11–0.66).

**Table 3:** Associations between biomarkers of inflammation and cardiometabolic complications in survivors of childhood acute lymphoblastic leukemia: crude and adjusted models.

Biomarker	Obesity		Insulin resistance		Pre-HTN/HTN		Dyslipidemia		MetS	
	OR (95% CI)	*p* value	OR (95% CI)	*p* value	OR (95% CI)	*p* value	OR (95% CI)	*p* value	OR (95% CI)	*p* value
	**Crude models**									
Adiponectin	**0.27 (0.14–0.52)**	** < 0.0001**	**0.21 (0.08–0.53)**	** < 0.001**	0.73 (0.30–1.81)	0.50	**0.36 (0.20–0.67)**	**0.001**	**0.08 (0.02–0.46)**	**0.004**
Leptin	**5.65 (2.76–11.6)**	** < 0.0001**	**6.09 (2.25–16.5)**	** < 0.001**	0.41 (0.15–1.09)	0.07	1.19 (0.64–2.23)	0.58	1.71(0.61–4.83)	0.32
Ratio leptin:adiponectin	**13.9 (6.09–31.7)**	** < 0.0001**	**16.6 (4.29–63.9)**	** < 0.001**	0.65 (0.26–1.67)	0.37	2.20 (1.16–4.17)	0.02	6.88 (1.71–27.7)	0.007
Resistin	1.04 (0.43–2.47)	0.94	2.48 (0.73–8.43)	0.15	1.03 (0.31–3.38)	0.97	1.81(0.77–4.24)	0.17	3.98 (0.89–17.8)	0.07
Visfatin	0.67 (0.36–1.28)	0.22	**0.25 (0.10–0.62)**	**0.003**	1.26 (0.5–3.19)	0.62	0.69 (0.37–1.29)	0.24	0.45 (0.16–1.32)	0.15
IL-6	1.79 (1.04–3.09)	0.04	0.90 (0.47–1.75)	0.76	1.18 (0.54–2.58)	0.68	1.25 (0.74–2.09)	0.40	1.80 (0.74–4.40)	0.20
TNF-α	1.86 (1.08–3.20)	0.03	1.81 (0.92–3.56)	0.09	1.02 (0.48–2.17)	0.96	1.10 (0.66–1.83)	0.72	2.85 (1.11–7.37)	0.03
PAI–1	**3.30 (1.64–6.62)**	** < 0.001**	3.23 (1.29–8.13)	0.01	1.66 (0.62–4.46)	0.32	2.23 (1.15–4.33)	0.02	2.94 (0.93–9.28)	0.07
CRP	**2.82 (1.57–5.07)**	** < 0.001**	**3.73 (1.87–7.47)**	** < 0.001**	0.97 (0.41–2.28)	0.95	**2.71 (1.52–4.83)**	** < 0.001**	**5.02 (2.03–12.4)**	** < 0.001**
	**Adjusted models**									
Adiponectin	**0.18 (0.09–0.38)**	** < 0.0001**	**0.17 (0.07–0.45)**	** < 0.001**	1.04 (0.41–2.66)	0.94	**0.34 (0.18–0.66)**	**0.002**	**0.07 (0.01–0.38)**	**0.002**
Leptin	**9.57 (3.41–26.9)**	** < 0.0001**	**13.2 (3.68–47.1)**	** < 0.0001**	0.99 (0.29–3.36)	0.99	1.70 (0.72–4.06)	0.23	3.21 (0.86–11.9)	0.08
Ratio leptin:aiponectin	**15.7 (6.20–39.7)**	** < 0.0001**	**20.6 (5.17–82.1)**	** < 0.0001**	1.14 (0.4–3.14)	0.81	2.89 (1.34–6.24)	0.007	**11.2 (2.58–48.7)**	**0.001**
Resistin	0.88 (0.34–2.30)	0.80	2.26 (0.65–7.85)	0.20	1.49 (0.42–5.32)	0.54	1.97 (0.74–5.21)	0.17	7.40 (1.29–42.5)	0.03
Visfatin	0.74 (0.38–1.45)	0.38	**0.27 (0.11–0.66)**	**0.004**	0.96 (0.36–2.54)	0.94	0.66 (0.34–1.32)	0.24	0.41 (0.14–1.25)	0.12
IL-6	1.72 (0.98–3.02)	0.06	0.88 (0.45–1.72)	0.71	1.23 (0.56–2.71)	0.61	1.22 (0.71–2.10)	0.47	1.78 (0.73–4.33)	0.21
TNF-α	**2.34 (1.31–4.18)**	**0.004**	2.13 (1.06–4.29)	0.03	0.83 (0.38–1.81)	0.65	1.22 (0.71–2.10)	0.46	3.40 (1.29–8.94)	0.01
PAI–1	**3.37 (1.61–7.05)**	**0.001**	3.14 (1.25–7.90)	0.02	1.78 (0.64–4.96)	0.27	1.99 (0.98–4.04)	0.06	2.40 (0.76–7.52)	0.13
CRP	2.21 (1.19**–**4.09)	0.01	**3.27 (1.58–6.79)**	**0.002**	1.53 (0.61**–**3.82)	0.37	**2.61 (1.40–4.86)**	**0.003**	**6.52 (2.38–17.9)**	< **0.001**

The crude and adjusted models were assessed between each biomarker and each cardiometabolic outcome. Models were adjusted for CRT exposure, age at diagnosis, time since diagnosis and sex. Odds ratio (non-corrected 95% CI) and p-value are indicated for each association. Significant associations are in boldface. Bonferroni-adjusted alpha = 0.05/number of biomarkers = 0.05/9 = 0.006. Metabolic syndrome was defined according to the International Diabetes Federation.

CI: confidence interval; CRP: C-reactive protein; CRT: cranial radiotherapy; HTN: arterial hypertension; IL-6: interleukin-6; MetS: metabolic syndrome; PAI-1: plasminogen activator inhibitor-1; TNF-α: tumor necrosis factor-α.

Among the biomarkers of oxidative stress measured, only oxidized-LDL (Ox-LDL) concentrations were associated with dyslipidemia (OR = 7.90; 95% CI 3.80–16.4). After correction for multiple testing, GSH, GPx, SOD and protein carbonyl levels were not associated with any cardiometabolic complication (Table 4). LBP, a biomarker of endotoxemia, was associated with obesity (OR = 2.03; 95% CI 1.13–3.65) and dyslipidemia (OR = 1.92; 95% CI 1.09–3.37) (Table 5). Levels of ICAM-1 were associated with obesity (OR = 2.15; 95% CI 1.21–3.82) and MetS (OR = 3.60; 95% CI 1.32–9.84) (Table 6).

**Table 4:** Associations between biomarkers of oxidative stress and cardiometabolic complications in survivors of childhood acute lymphoblastic leukemia: crude and adjusted models.

Biomarker	Obesity		Insulin resistance		Pre-HTN/HTN		Dyslipidemia		MetS	
	OR (95% CI)	*p* value	OR (95% CI)	*p* value	OR (95% CI)	*p* value	OR (95% CI)	*p* value	OR (95% CI)	*p* value
	**Crude models**									
GSH	0.55 (0.32–0.95)	0.03	0.71 (0.37–1.39)	0.32	1.08 (0.50–2.33)	0.85	0.79 (0.47–1.32)	0.36	1.35 (0.56–3.29)	0.51
GPx	0.83 (0.49–1.42)	0.50	0.80 (0.41–1.55)	0.50	1.57 (0.73–3.38)	0.25	1.78 (1.06–2.97)	0.03	1.22 (0.52–2.91)	0.65
Ox-LDL	1.31 (0.70–2.44)	0.40	1.39 (0.63–3.06)	0.41	0.82 (0.32–2.05)	0.66	**7.89 (3.97–15.7)**	** < 0.0001**	3.08 (1.00–9.55)	0.05
Protein carbonyls	1.04 (0.43–2.47)	0.94	1.72 (0.53–5.58)	0.37	0.49 (0.14–1.69)	0.26	1.26 (0.54–2.92)	0.60	1.52 (0.42–5.57)	0.53
SOD	1.24 (0.65–2.36)	0.52	1.09 (0.49–2.42)	0.84	0.80 (0.32–2.03)	0.64	0.90 (0.48–1.69)	0.75	0.76 (0.27–2.10)	0.59
mtDNA	1.14 (0.65–2.00)	0.64	0.89 (0.44–1.80)	0.74	0.86 (0.39–1.89)	0.71	1.09 (0.64–1.86)	0.75	1.59 (0.61–4.17)	0.34
	**Adjusted models**									
GSH	0.51 (0.29–0.91)	0.02	0.72 (0.36–1.42)	0.34	1.35 (0.61–2.99)	0.46	0.88 (0.51–1.51),	0.65	1.62 (0.65–4.00)	0.30
GPx	0.84 (0.48–1.46)	0.53	0.80 (0.41–1.57)	0.52	1.56 (0.71–3.43)	0.27	1.98 (1.15–3.42)	0.01	1.30 (0.55–3.09)	0.55
Ox-LDL	1.36 (0.71–2.62)	0.35	1.36 (0.61–3.02)	0.46	0.66 (0.25–1.71)	0.39	**7.90 (3.80–16.4)**	** < 0.0001**	2.87 (0.93–8.86)	0.07
Protein carbonyls	0.72 (0.28–1.90)	0.51	1.42 (0.42–4.78)	0.58	0.57 (0.16–2.10)	0.40	1.02 (0.40–2.63)	0.96	1.73 (0.42–7.20)	0.45
SOD	1.11 (0.55–2.21)	0.78	0.97 (0.42–2.23)	0.94	1.17 (0.43–3.17)	0.76	0.88 (0.43–1.78)	0.72	0.82 (0.29–2.39)	0.72
mtDNA	1.27 (0.71–2.25)	0.43	0.97 (0.48–1.98)	0.94	0.78 (0.35–1.75)	0.55	1.22 (0.70–2.13)	0.49	1.82 (0.69–4.77)	0.22

The crude and adjusted models were assessed between each biomarker and each cardiometabolic outcome. Models were adjusted for CRT exposure, age at diagnosis, time since diagnosis and sex. Odds ratio (non-corrected 95% CI) and p-value are indicated for each association. Significant associations are in boldface. Bonferroni-adjusted alpha = 0.05/number of biomarkers = 0.05/6 = 0.008. Metabolic syndrome was defined according to the International Diabetes Federation.

CI: confidence interval; CRT: cranial radiotherapy; GPx: glutathione peroxidase; GSH: glutathione; HTN: arterial hypertension; MetS: metabolic syndrome; mtDNA: mitochondrial DNA; Ox-LDL: oxidized low-density lipoprotein; SOD: superoxide dismutase.

**Table 5:** Associations between biomarkers of endotoxemia and cardiometabolic complications in survivors of childhood acute lymphoblastic leukemia: crude and adjusted models.

Biomarker	Obesity		Insulin resistance		Pre-HTN/HTN		Dyslipidemia		MetS	
	OR (95% CI)	*p* value	OR (95% CI)	*p* value	OR (95% CI)	*p* value	OR (95% CI)	*p* value	OR (95% CI)	*p* value
	**Crude models**									
LPS	0.60 (0.29–1.24)	0.17	0.91 (0.36–2.31)	0.85	0.88 (0.31–2.54)	0.82	0.80 (0.40–1.62)	0.53	0.39 (0.11–1.45)	0.16
LBP	**2.59 (1.48–4.51)**	**0.001**	**2.26 (1.13–4.51)**	**0.021**	0.74 (0.35–1.59)	0.45	**2.18 (1.30–3.67)**	**0.003**	2.75 (1.07–7.11)	0.04
	**Adjusted models**									
LPS	0.58 (0.27–1.25)	0.16	0.88 (0.34–2.27)	0.80	0.90 (0.29–2.75)	0.85	0.83 (0.39–1.77)	0.62	0.44 (0.12–1.59)	0.21
LBP	**2.03 (1.13–3.65)**	**0.019**	1.77 (0.86–3.66)	0.12	0.99 (0.44–2.23)	1.00	**1.92 (1.09–3.37)**	**0.024**	2.60 (0.96–7.06)	0.06

The crude and adjusted models were assessed between each biomarker and each cardiometabolic outcome. Models were adjusted for CRT exposure, age at diagnosis, time since diagnosis and sex. Odds ratio (non-corrected 95% CI) and p-value are indicated for each association. Significant associations are in boldface. Bonferroni-adjusted alpha = 0.05/number of biomarkers = 0.05/2 = 0.025. Metabolic syndrome was defined according to the International Diabetes Federation.

CI: confidence interval; CRT: cranial radiotherapy; HTN: arterial hypertension; LBP: lipopolysaccharide-binding protein; LPS: lipopolysaccharide; MetS: metabolic syndrome.

**Table 6:** Associations between biomarkers of endothelial function and cardiometabolic complications in survivors of childhood acute lymphoblastic leukemia: crude and adjusted models.

Biomarker	Obesity		Insulin resistance		Pre-HTN/HTN		Dyslipidemia		MetS	
	OR (95% CI)	*p* value	OR (95% CI)	*p* value	OR (95% CI)	*p* value	OR (95% CI)	*p* value	OR (95% CI)	*p* value
	**Crude models**									
ICAM-1	**2.11 (1.22–3.65)**	**0.007**	2.00 (1.01–3.96)	0.05	1.00 (0.47–2.13)	1.000	1.83 (1.10–3.07)	0.02	**3.51 (1.30–9.53)**	**0.014**
VCAM-1	0.60 (0.35–1.02)	0.06	0.63 (0.32–1.24)	0.18	1.16 (0.54–2.47)	0.70	0.58 (0.35–0.98)	0.04	0.83 (0.35–1.98)	0.68
E-Selectin	2.44 (0.96–6.20)	0.06	1.03 (0.31–3.42)	0.96	0.71 (0.21–2.40)	0.58	1.42 (0.59–3.47)	0.44	1.62 (0.44–6.00)	0.47
	**Adjusted models**									
ICAM-1	**2.15 (1.21–3.82)**	**0.009**	1.91 (0.95–3.84)	0.07	1.09 (0.50–2.38)	0.81	1.82 (1.06–3.13)	0.03	**3.60 (1.32–9.84)**	**0.013**
VCAM-1	0.86 (0.47–1.57)	0.62	0.84 (0.40–1.77)	0.65	0.81 (0.35–1.88)	0.62	0.79 (0.44–1.42)	0.43	1.30 (0.50–3.40)	0.583
E-Selectin	3.41 (1.15–10.09)	0.03	1.10 (0.33–3.72)	0.88	0.55 (0.15–2.04)	0.37	1.65 (0.60–4.57)	0.33	1.59 (0.40–6.25)	0.511

The crude and adjusted models were assessed between each biomarker and each cardiometabolic outcome. Models were adjusted for CRT exposure, age at diagnosis, time since diagnosis and sex. Odds ratio (non-corrected 95% CI) and p-value are indicated for each association. Bonferroni-adjusted alpha = 0.05/number of biomarkers = 0.05/3 = 0.017. Metabolic syndrome was defined according to the International Diabetes Federation.

CI: confidence interval; CRT: cranial radiotherapy; HTN: arterial hypertension; ICAM-1: intercellular adhesion molecule-1; MetS: metabolic syndrome; VCAM-1: vascular cell adhesion molecule-1.

Among the disturbances that define dyslipidemia, only an association with low HDL-C was observed. High LBP levels were associated with low HDL-C (OR = 3.26; 95% CI 1.37–7.73) (Supplementary Table S2). No other association was found when the associations between the biomarkers and the risk of having high triglycerides, high LDL-C, and low HDL-C were analyzed individually (Supplementary Tables S3 to S5).

### Structural equation models to assess LBP-inflammation-cardiometabolic complication relationships

**Figure 1:**
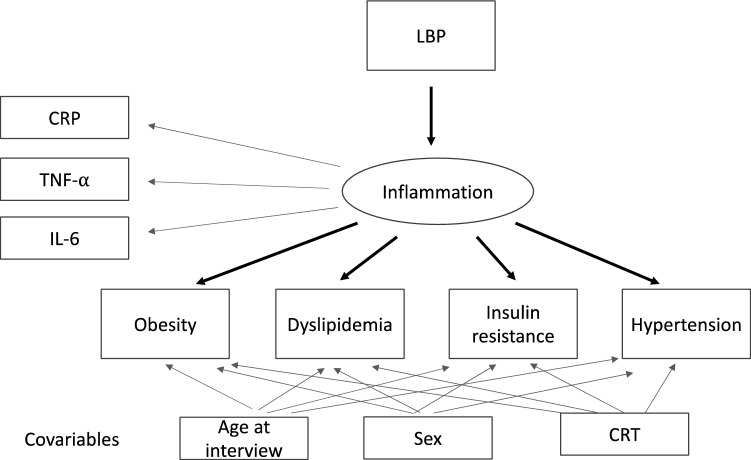
Path diagram corresponding to structural equation model between LBP (biomarker of endotoxemia), inflammation as the latent variable (derived from CRP, TNF-α and IL-6) and cardiometabolic outcomes. The conventional rules of SEM visualization were applied.

**Figure 2:**
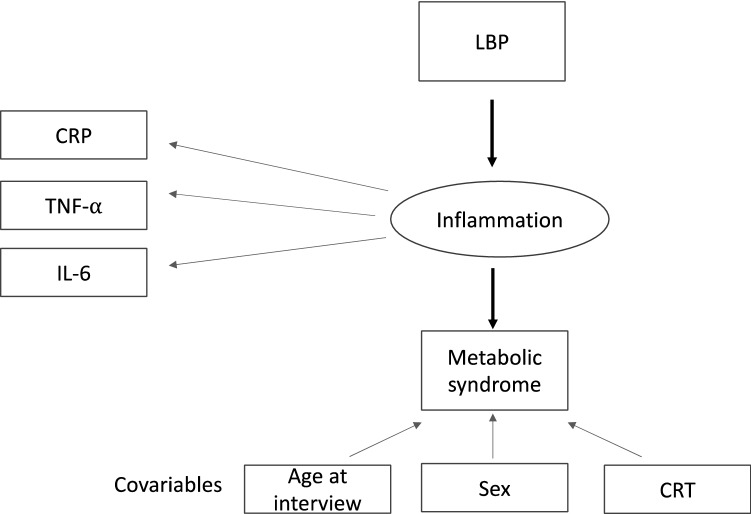
Path diagram corresponding to structural equation model between LBP (biomarker of endotoxemia), inflammation as the latent variable (derived from CRP, TNF-α and IL-6) and metabolic syndrome. The conventional rules of SEM visualization were applied.

For two SEM considered (see Figs. 1 and 2), estimated factor loadings of CRP, TNF-α and IL-6 for the latent variable *inflammation*, as well as the path coefficients reflecting the relationships between LBP, *inflammation* and the cardiometabolic outcomes are presented in Tables 7 and 8. As indicated by the goodness-of-fit measures, the models hypothesized generally appeared to fit well the data^48,49^. Estimated path coefficients representing relationships between LBP, *inflammation* and cardiometabolic outcomes indicated expected significant directional relationships with the exception of hypertension (Table 7). The standardized regression coefficients between LBP, inflammation and MetS were also statistically significant (Table 8). Of note, the covariables *age at interview* and *sex* were associated with dyslipidemia and hypertension respectively (Table 7). The covariable exposition to CRT was not associated with cardiometabolic outcomes (Tables 7 and 8).

**Table 7:** SEM estimates of the associations between LBP, *inflammation*, obesity, dyslipidemia, insulin resistance and pre-HTN/HTN.

	Non standardized estimates	Standardized estimates	
	Estimate (95% CI)	Estimate (95% CI)	*p* value
**Loadings of the biomarkers CRP, TNF-α and IL-6 for the latent variable inflammation**			
CRP	1.000	0.871 (0.761, 0.981)	** < 0.001**
TNF-α	0.012 (− 0.087, 0.110)	0.012 (− 0.086, 0.109)	0.82
IL-6	0.060 (0.031, 0.089)	0.345 (0.219, 0.472)	** < 0.001**
**Regression of inflammation on LBP**			
LBP	0.304 (0.245, 0.363)	0.714 (0.604, 0.823)	** < 0.001**
**Regression of obesity on inflammation, sex, age at interview, CRT**			
*Inflammation*	0.097 (0.041, 0.153)	0.324 (0.216, .552)	** < 0.001**
Sex (M vs. F)	− 0.248 (− 0.607, 0.111)	− 0.117 (− 0.278, 0.049)	0.17
Age at interview	0.009 (− 0.019, 0.038)	0.056 (− 0.113, 0.223)	0.52
CRT (yes vs. no)	0.185 (− 0.198, 0.567)	0.086 (− 0.088, 0.256)	0.34
**Regression of dyslipidemia on inflammation, sex, age at interview, CRT**			
*Inflammation*	0.082 (0.024, 0.139)	0.274 (0.099, 0.448)	** < 0.01**
Sex (M vs. F)	0.197 (− 0.168, 0.561)	0.093 (− 0.079, 0.266)	0.29
Age at interview	0.031 (0.001, 0.062)	0.188 (0.011, 0.365)	**0.04**
CRT (yes vs. no)	0.125 (− 0.247, 0.498)	0.058 (− 0.115, 0.231)	0.51
**Regression of insulin resistance on inflammation, sex, age at interview, CRT**			
*Inflammation*	0.115 (0.056, 0.173)	0.381 (0.212, 0.549)	** < 0.001**
Sex (M vs. F)	− 0.127 (− 0.544, 0.290)	− 0.060 (− 0.255, 0.135)	0.55
Age at interview	0.006 (− 0.032, 0.044)	0.037 (− 0.187, 0.261)	0.75
CRT (yes vs. no)	0.253 (− 0.180, 0.686)	0.117 (− 0.079, 0.313)	0.24
**Regression of pre-HTN/HTN on inflammation adjusted for sex, age at interview, CRT**			
*Inflammation*	0.014 (− 0.085, 0.113)	0.046 (− 0.276, 0.367)	0.78
Sex (M vs. F)	0.801 (0.239, 1.363)	0.371 (0.142, 0.600)	** < 0.01**
Age at interview	− 0.007 (− 0.050, 0.035)	− 0.043 (− 0.291, 0.205)	0.73
CRT (yes vs. no)	0.234 (− 0.270, 0.737)	0.106 (− 0.121, 0.333)	0.36
**Goodness of fit measures**: CFI = 0.997, TLI = 0.997, RMSEA (90% CI) = 0.010 (0.000, 0.056), SRMR = 0.090			

CRT: cranial radiotherapy; CFI: Comparative Fit Index; HTN: hypertension; LBP: LPS binding protein; RMSEA: Root Mean Square Error of Approximation; SRMR: Standardized Root Mean Square Residual; TLI: Tucker-Lewis Index.

*p* values of non-standardized estimates are not shown as they are almost identical to *p* values of standardized estimates.

**Table 8:** SEM estimates of the associations between LBP, *inflammation* and metabolic syndrome.

	Non standardized estimates	Standardized estimates	
	Estimate (95% CI)	Estimate (95% CI)	*p* value
**Loadings of the biomarkers CRP, TNF-α and IL-6 for the latent variable inflammation**			
CRP	1.000	0.944 (0.767, 1.122)	** < 0.001**
TNF-α	0.002 (− 0.072, 0.076)	0.002 (− 0.076, 0.081)	0.95
IL-6	0.051 (0.019, 0.083)	0.322 (0.181, 0.463)	** < 0.001**
**Regression of inflammation on LBP**			
LBP	0.297 (0.238, 0.355)	0.649 (0.502, 0.796)	** < 0.001**
**Regression of metabolic syndrome on inflammation adjusted for sex, age at interview, CRT**			
*Inflammation*	0.099 (0.031, 0.168)	0.345 (0.153, 0.537)	** < 0.001**
Sex (M vs. F)	0.397 (− 0.143, 0.937)	0.182 (− 0.055, 0.418)	0.13
Age at interview	0.045 (− 0.004, 0.093)	0.254 (− 0.009, 0.517)	0.06
CRT (yes vs. no)	0.131 (− 0.513, 0.774)	0.059 (− 0.230, 0.347)	0.69
**Goodness of fit measures**: CFI = 1.000, TLI = 1.042, RMSEA (90% CI) = 0.000 (0.000, 0.043), SRMR = 0.025			

CRT: cranial radiotherapy; CFI: Comparative Fit Index; LB: LPS binding protein; RMSEA: Root Mean Square Error of Approximation; SRMR: Standardized Root Mean Square Residual; TLI: Tucker-Lewis Index.

*p* values of non-standardized estimates are not shown as they almost identical to *p* values of standardized estimates.

## Discussion

We found associations between several inflammatory biomarkers and obesity, dyslipidemia and insulin resistance in a cohort of adolescent and young adult survivors of cALL. Our analyses support the hypothesis of a directional relationship between LBP, a biomarker of endotoxemia, *inflammation* and cardiometabolic outcomes. These results are in agreement with the hypothesis that, in cALL survivors, endotoxemia triggers a pro-inflammatory state that is associated with the development of cardiometabolic complications.

We found that high TNF-α levels were associated with the risk of obesity. TNF-α contributes to metabolic dysregulation by impairing lipid storage and oxidative capacity of adipose tissues^50^. TNF-α can impact whole body insulin sensitivity through increased free fatty acids and altered adipokine production^50^. Our finding corroborates a large body of literature showing a strong correlation between TNF-α plasma levels and obesity [reviewed in^51^], as well as its relationship with BMI in obese children^52,53^ and adolescents^54^.

Our analyses also revealed that high CRP levels are associated with the risk of insulin resistance, dyslipidemia and MetS. CRP is an acute-phase protein secreted by the liver and the adipose tissue in response to pro-inflammatory cytokines (e.g. TNF-α and IL-6). It is a sensitive marker of systemic inflammation^55^ and has been associated with cardiometabolic risk^56^. Consistent with our results, many studies reported increased plasma CRP in cALL survivors compared to controls^7–9,29^. CRP levels were associated with the MetS in this population^8^.

Our results showed altered adipokine levels in relation to cardiometabolic outcomes. Adjusted model showed that higher leptin-adiponectin ratio was associated with an elevated risk of obesity, insulin resistance and low HDL-C. There are compelling data that emphasize the associations between adiponectin, leptin and cardiometabolic complications in cALL survivors^19–21,57–60^. In adolescent survivors, high leptin-adiponectin ratio was associated with MetS^20^. Particularly, visceral adiposity was associated with disturbed adipokine balance (i.e., increased leptin and decreased adiponectin)^61,62^, chronic low-grade inflammation^63^ and dyslipidemia as characterized by decreased HDL-C levels. Our results support the reported positive association between plasma adiponectin levels and HDL-C^64^. Adiponectin regulates HDL-C concentration by reducing HDL-C catabolism^65^ and contributes to the inhibition of hepatic lipase activity^65^, an enzyme that hydrolyzes triglycerides and phospholipids contained in HDL particles. In our study, high leptin-adiponectin ratio was associated with increased risk of insulin resistance. In line with our results, HOMA-IR has been associated with higher leptin-adiponectin ratio in both men and women^19^ and in women only^58^.

We also found that high leptin levels were strongly associated with the risk of obesity and insulin resistance. This finding corroborates the presence of long-term hyperleptinemia in cALL survivors^19,21^ that may disrupt communication with the brain and, ultimately, energy usage and insulin signalling^66^. Our results also emphasized the association between low visfatin levels and the risk of insulin resistance. The role of visfatin in insulin resistance is still controversial [reviewed in^67^]. Visfatin was reported to have insulin-like activity and to bind the insulin receptor, thereby lowering blood glucose levels^68^. In mice, acute administration of visfatin lowered blood glucose levels^68^. Interestingly, transient overexpression of visfatin enhanced its plasma level, improved insulin sensitivity and had hypocholesterolemic effects in normal-chow rats and rats on high-fat diet^69,70^. However, another study found that visfatin does not have insulin-mimetic effects in mice but rather functions as an extracellular NAD biosynthetic enzyme critical for glucose-stimulated insulin secretion by pancreatic β-cells^71^. Although studies have provided evidence that visfatin is a biomarker for cALL remission^72^ and bone mineral density of cALL survivors^73^, no prior study had yet examined the relationship between visfatin levels and cardiometabolic risk factors in the context of survivorship. In parallel, we have highlighted the association between high PAI-1 levels, a physiological regulatory protein of the fibrinolytic system, and the risk of obesity in our cohort. In line with our findings, a study reported increased plasma PAI-1 levels in cALL survivors compared to controls^74^, but the association with obesity was not assessed.

Clinical studies have demonstrated that serum LPS levels were closely related to obesity^75–77^ and insulin resistance^76,78,79^. While our study did not reveal an association, we cannot exclude that this negative result could be the result of the short half-life of LPS^80^. LBP is considered a biomarker of plasma endotoxin exposure because its half-life (24 h) is longer than of LPS (< 8 min in mice and up to 3 h in humans)^81^. Hence, it has been used as a proxy to assess chronic endotoxemia^82^. We found that higher LBP levels were associated with an increased risk of obesity, insulin resistance and low HDL-C in cALL survivors. LBP participates in the LPS-mediated inflammatory response, facilitating the binding of LPS to toll-like receptor 4 (TLR4)^83^. TLR4 activation leads to increase transcription pro-inflammatory genes that promote the initiation of cytokine signalling cascades^83^, reactive oxygen species production and insulin desensitization^84^. TLR4 activation by LPS and LBP complex in insulin target cells can directly dampen insulin action through mechanisms involving cytokine and pro-inflammatory kinases JNK, IKK and p38^84^. LBP also acts as a lipid transfer protein by catalysing the transfer of LPS-sCD14 complexes to HDL particles^85^. Our findings corroborate other studies in which LBP levels were strongly associated with lipid abnormalities^75,86–89^. They are also in agreement with two prospective studies showing associations between higher LBP levels and the risk of insulin resistance and impaired fasting glucose in men^90^, and with the risk of MetS in men and women^89^. Similarly, cross-sectional studies highlighted a relationship between higher LBP levels and insulin resistance in adolescent men and women^91^ or in adult men^75,92^. Also, in our study, the SEM analysis supports that the relationships between endotoxemia and obesity, insulin resistance, dyslipidemia and MetS are mediated by *inflammation*. This indicates that circulating LBP is a relevant biomarker for systemic inflammation in cALL survivors.

Among the biomarkers of OxS examined, associations were found only for ox-LDL with dyslipidemia and having low HDL-C. There has been a growing body of evidence suggesting that plasma ox-LDLs are increased in pathologic conditions linked to cardiovascular diseases including insulin resistance, obesity and MetS^93,94^. As for endothelial function, only ICAM-1 levels were associated with the risk of obesity and MetS. A recent study reported increased plasma ICAM-1 levels in 64 cALL survivors (median age of 15 years) compared to 36 controls^29^. Conversely to our findings, a small study by Barbosa-Cortés et al. did not find any association between circulating ICAM-1 levels and the prevalence of the MetS in cALL or lymphoma survivors^20^. These discrepancies with our study may be due, in part, to different patients’ characteristics (median age of 12.1 vs. 21.8 years), CRT exposure (32.7 vs. 59.4%) and number of participants with MetS (n = 7 vs. n = 22). As a matter of fact, it was reported that ICAM-1 levels can be influenced by age, insulin resistance and other inflammatory conditions^95^. We did not report associations between VCAM-1 or E-selectin and cardiometabolic complications. Elsewhere, a small study comprising of 27 cALL survivors (median age of 20 years) reported high VCAM-1 levels compared to 20 controls^30^, but the association with cardiometabolic complications was not investigated. In the general population, no evidence of a positive association of VCAM-1 and cardiometabolic outcomes has been reported^96–98^. However, it was suggested that VCAM-1 is expressed primarily at an advanced stage of atherosclerosis^98^ whereas ICAM-1 is a general marker of a pro-inflammatory state in healthy population. Besides, E-selectin was found correlated with obesity and insulin resistance among obese subjects^99,100^ and to be a good predictor for insulin resistance in women^101^. In the context of cALL survivorship, no prior study has examined the relationship between E-selectin and cardiometabolic complications, despite the important roles played by this protein in endothelial function.

Finally, no significant association was found between the biomarkers and hypertension. Conversely, two studies in cALL and lymphoma survivors found that low levels of adiponectin were associated with hypertension^20,22^. These different findings could be explained by the fact that our study was restricted to survivors of cALL and by the low prevalence of hypertension in our cohort (12%) compared to the others (27% and 54%).

The strengths of our study include a broad panel of blood biomarkers of endotoxemia, inflammation, oxidative stress and endothelial function in a well-characterized cohort of cALL survivors. We used the SEM, a multivariate statistical analysis technique, in addition to the conventional regression analysis. This allows to simultaneously test the relationships between the different variables potentially explaining the development of the cardiometabolic complications. Limitations include the monocentric study design and the absence of a healthy control group. Additionally, as our study population was Caucasian, our results may not be generalizable to other ethnic groups as, in epidemiological studies, plasma levels of inflammatory biomarkers associated with cardiometabolic diseases can differ by ethnic group^102^. Although we did adjust a priori for four selected confounding variables, residual confounding by other factors may bias the results. The modest size of our cohort (n = 246, corresponding to the maximal sample size) may have limited our ability to detect weak associations, especially given that sample sizes available for analyses depended on biomarkers. In general, we acknowledge that our study may have generated both false-negative and false-positive findings and will need to be replicated in larger samples. Since structural equation modeling is very sensible to sample size^103^ our results should be interpreted with caution. Furthermore, there is no cutoff for fit indices to evaluate the goodness of fit for a model including binary variables^104,105^. Hence, the cutoff used to evaluate the goodness of fit of our models were defined for models with continuous variables, a limit that has to be considered.

## Conclusions

This study revealed significant associations between plasma biomarkers of visceral inflammation, endotoxemia and endothelial function and late occurring cardiometabolic adverse effects in cALL survivors. It also highlights the relationship between LBP, a protein related to metabolic endotoxemia, *inflammation* and the presence of cardiometabolic complications. Identification of biomarkers and biological mechanisms could open new avenues for prevention strategies to minimize the long-term sequelae, improve patient follow-up and ultimately optimize the quality of life of this high-risk population.

## Material and methods

### Study population and protocol

Participants survivors of cALL included in this study (n = 246) were recruited as part of the PETALE study (Sainte-Justine University Health Center (SJUHC), Quebec, Canada). The study design and cohort characteristics are described in^106^. Briefly, subjects enrolled in the PETALE study were treated for cALL at SJUHC with the Dana Farber Cancer Institute protocols 87–01 to 05–01^107^. Survivors less than 19 years old at diagnosis, more than 5 years post diagnosis and free of relapse were invited to participate. At interview, participants completed a core laboratory assessment as well as anthropometric and clinical evaluations. Demographic characteristics relevant for the following analyses are outlined in Table 1. The study was approved by the Institutional Review Board of SJUHC and investigations were carried out in accordance with the principles of the Declaration of Helsinki. Written informed consent was obtained from study participants or parents/guardians.

### Assessment of cardiometabolic complications

Participants were assessed for cardiometabolic outcomes, specifically obesity, insulin resistance, pre-HTN and HTN and dyslipidemia (cut-off values presented in Supplementary Table S1). The prevalence of cardiometabolic complications in this cohort has been previously described^4^. Briefly, obesity was determined by the presence of at least one of two factors: obese according to body mass index (BMI)^108,109^ and/or high waist circumference^110,111^. Insulin resistance measured in fasting plasma and defined by the presence of at least two of three factors: glucose > 6.1 mmol/L; and/or; glycated hemoglobin > 6.0%; and/or; homeostasis model assessment [HOMA-IR, insulin (mIU/L) × glucose (mmol/L)/22.5)] ≥ 2.86 (adults)^112^ or ≥ 95th percentile (children)^113^. Pre-HTN and HTN were determined according to current recommendations in adults (normal < 130/85 mmHg; pre-HTN: ≥ 130/85 and < 140/90 mmHg; HTN ≥ 140/90 mmHg)^114^ and in children (normal: < 90th percentile; pre-HTN: ≥ 90th and < 95th percentile and HTN ≥ 95th percentile according to age and height)^115^. Participants who were taking drugs to treat hypertension were also considered hypertensive. Dyslipidemia was determined in fasting plasma by the presence of at least one of three factors: high LDL-C; high triglycerides and/or low HDL-C according to cut-off values for age and sex^116,117^. MetS was defined according to the International Diabetes Federation^118^. For participants 16 years and older, we considered: having waist circumference ≥ 94 cm in men and ≥ 80 cm in women, plus any two of the following factors: (i) triglycerides ≥ 1.70 mmol/L or on drug treatment; (ii) HDL-C < 1.03 mmol/L in men and < 1.3 mmol/L in women or on therapy; (iii) systolic ≥ 130 mmHg or diastolic ≥ 85 mmHg or on treatment and; (iv) fasting glucose ≥ 5.6 mmol/L. For children 10 to 16 years, MetS was defined by waist circumference ≥ 90th percentile plus any two of: (i) triglycerides ≥ 1.70 mmol/L; (ii) HDL-C < 1.03 mmol/L; (iii) systolic ≥ 130 mmHg or diastolic ≥ 85 mmHg and; (iv) fasting glucose ≥ 5.6 mmol/L.

### Quantification of biomarkers

Overnight fasting peripheral blood samples were collected in EDTA tubes that were kept on ice until centrifugation. Plasma was separated by low speed centrifugation (2200*g*, 20 min) at 4 °C within 45 min of collection and stored at − 80 °C until analysis. White blood cells were isolated and stored at − 80 °C until analysis. Red blood cells (RBCs) were washed with saline, butylated hydroxytoluene (10 µl/ml) (Sigma-Aldrich, St. Louise, MO, USA) was added and RBCs were stored at − 80 °C until analysis.

#### Biomarkers of inflammation

Commercial ELISA kits were used to measure the following biomarkers in fasting plasma: adiponectin (#BMS2032), resistin (#BMS2040) and plasminogen activator inhibitor-1 (PAI-1) (#BMS2033) (Thermo Fisher Scientific, Waltham, MA, USA), leptin (# EZHL-80SK, EMD Millipore, Burlington, MA, USA) and visfatin (#EIA-VIS, RayBiotech, GA, USA). High sensitivity CRP was measured in fasting serum by immunoturbidimetry. IL-6 and TNF-α were measured in fasting plasma using the multiplex ELISA V-Plex Pro-inflammatory Panel I according to the manufacturer’s instructions (MesoScale Discovery, Rockville, MD, USA).

#### Biomarkers of oxidative stress

SOD activity was measured in plasma by the method of xanthine/xanthine oxidase using kits from Caymen Chemical (#706002, Ann Arbor, MI, USA). GPx activity was measured in RBCs by measuring consumed NADPH using kits from Caymen Chemical (#703102, Ann Arbor, MI, USA). Ox-LDL and protein carbonyls were measured using ELISA kits from Mercodia AB (#10-1143-01, Uppsala, Sweden) and Cell Biolabs Inc. (#STA-310, San Diego, CA, USA), respectively. Total glutathione was measured in RBCs by spectrophotometry with the GSH recycling method^119,120^. The intra-assay CVs for SOD, GPx, Ox-LDLs, GSH were 24.2%, 11.4%, 9.5% and 10.8%, respectively. Mitochondrial DNA was determined by Quantitative real-time PCR. Genomic DNA was extracted from white blood cells using the Purelink Genomic DNA kit (Thermo Fisher Scientific) following manufacturer’s instructions. DNA was quantified using a spectrophotometer and concentrations were adjusted to 10 ng/ml. qPCR for human mitochondria-encoded NADH dehydrogenase 1 (*MT-ND1*) and hemoglobin subunit beta (*HBB*) expression analysis was performed using Taqman gene expression probes #*Hs02596873_s1* and *#Hs00758889_s1*, respectively. Transcript expression was determined with the 7500 Fast Real-Time PCR System (Applied Biosystems, Foster City, CA, USA). Expression levels were measured by Relative Quantity (RQ, 2^−ΔΔCt^), where *HBB* expression served as endogenous control for normalization^121^.

#### Biomarkers of endotoxemia

Levels of LPS and LBP were measured in fasting plasma using commercial ELISA kits from Elabscience (#E-EL-0025, Houston, TX, USA) and Cell Sciences (#CKM043, Newburyport, MA, USA), respectively. The intra-assay CVs for LPS and LBP were 15.7% and 8.1%, respectively.

#### Biomarkers of endothelial function

Levels of E-selectin were measured using kits from RayBiotech (#ELH-Eselectin, Norcross, GA, USA). ICAM-1 and VCAM-1 were measured in fasting plasma with V-PLEX assay kits (Meso-Scale Discovery, Rockville, MD, USA). All experiments were achieved following manufacturers’ protocols and all readings were performed using the DTX800/800 Multimode microplate reader (Beckman Coulter, Brea, CA, USA).

### Statistical analyses

The biomarkers were dichotomized by the median, with the exception of CRP that was dichotomized by comparing normal (≤ 3 mg/L) to high (> 3 mg/L) levels. The association was assessed between each biomarker and each cardiometabolic outcome using a logistic regression analysis with the targeted biomarker as the independent variable. Analyses were performed without adjustment (crude models) and with adjustment for CRT exposure, age at diagnosis, time since diagnosis and sex (adjusted models). Firth’s penalized maximum likelihood estimation procedure was used to mitigate problems of quasi-complete separation of data points encountered in standard logistic regression analyses^122,123^. Nominal significance level α was set to 0.05. Biomarkers were categorized according to their functional pathways. To consider multiple testing, Bonferroni-adjusted significance level α_adj_ was calculated within the following groups of biomarkers: inflammation, oxidative stress, endotoxemia and endothelial function (α_adj_ = α/k, where k is the number of biomarkers in each category: inflammation: k = 9; oxidative stress: k = 6; endotoxemia: k = 2; and endothelial function: k = 3). Analyses were performed using SAS software, version 9.4 (SAS Institute, Cary, NC, USA).

We examined the relationship between LBP (endotoxemia), inflammation and metabolic outcomes using structural equation models (SEM). A SEM estimates all model parameters simultaneously and thus assesses the strength of a particular relationship within the context of a model that can include both measured and constructed (latent) variables ^124^. We hypothesized that LBP was associated with the cardiometabolic outcomes through full mediation by the latent (unobserved) variable *inflammation* inferred from the observed biomarkers CRP, TNF-α and IL-6. In the SEM, the estimation process aims to minimize the difference between the sample- and model-implied variance–covariance matrices. The latent variable was estimated by analysing the variance and covariance of the biomarkers (CRP, TNF-α and IL-6). The loading biomarker CRP was fixed to 1 and the residual covariances of the latent variables were set at zero. Two structural models were considered. In the first model, the studied cardiometabolic outcomes were obesity, dyslipidemia, insulin resistance and HTN. In the second model, MetS was the only outcome studied. In the two models, the outcome regressions were adjusted for CRT exposure, age at interview and sex. Figures 1 and 2 present the path diagrams corresponding to models 1 and 2, respectively. The conventional rules of SEM visualization were applied^125^. The diagonally weighted least squares (DWLS) estimator was used to handle the studied binary outcomes^105^. Standardized parameter estimates were presented in addition to their non standardized counterparts in order to compare the relative impact of variables measured on the different scales^126^. Model fit was evaluated using Comparative Fit Index (CFI), TLI (Tucker–Lewis index), Root Mean Square Error of Approximation (RMSEA), and Standardized Root Mean Square Residual (SRMR). The conventional cutoffs indicating a good model–data fit are CFI > 0.95, TLI > 0.95, RMSEA < 0.06, and SRMR < 0.08^127^, but some recent studies have cautioned against their applying to ordered categorical data^104,105^.The SEM analyses were performed using R package Lavaan version 0.6-6.

## Supplementary Information

Supplementary Information.

## Data Availability

The data generated and analysed during the current study are not publicly available due to confidentiality reasons but are available from the corresponding author on reasonable request.
