# Supplementary material for: Biomarkers of cardiometabolic complications in survivors of childhood acute lymphoblastic leukemia
Source: Sci Rep. 2020 Dec 9;10:21507. doi: 10.1038/s41598-020-78493-x (PMC7726154; doi:10.1038/s41598-020-78493-x)
Supplement: Supplementary file 1 — Supplementary Information. [file 41598_2020_78493_MOESM1_ESM.pdf]

Supplementary Tables

Biomarkers of Cardiometabolic Complications in Survivors of Childhood Acute Lymphoblastic Leukemia

Sophia Morel[1,5], Pauline Léveillé[1,2], Mariia Samoilenko[1,3], Anita Franco[1], Jade England[1,4], Nicolas Malaquin[6], Véronique Tu[6], Guillaume B. Cardin[6], Simon Drouin[1], Francis Rodier[6,7], Sarah Lippé[1,2], Maja Krajinovic[1,4], Caroline Laverdière[4], Daniel Sinnett[4], Geneviève Lefebvre[3], Emile Levy[1,5], Valérie Marcil[1,5*]

Supplementary Table S1: Cardiometabolic outcomes cut-off values.

Risk factor	Adults	Children
**Obesity**		
High BMI	≥30 kg/m^2	≥97th percentile
High WC	≥102 cm (men) and ≥88 cm (women)	≥95th percentile
**Insulin resistance**		
High blood fasting glucose	≥6.1 mmol/L	≥6.1 mmol/L
High glycated hemoglobin	≥6% and <6.5%	≥6% and <6.5%
High HOMA-IR	≥2.86	≥95th percentile of a French-Canadian population
**Hypertension**		
Pre-hypertension	≥130/85 and <140/90 mmHg	≥90th and <95th percentile for age and height
Hypertension	≥140/90 mmHg or taking medication	≥95th percentile for age and height or taking medication
**Dyslipidemia**		
High TG	≥1.7 mmol/L	≥1.47 mmol/L
High LDL-C	≥3.4 mmol/L	≥3.36 mmol/L
Low HDL-C	<1.03 (men) and <1.3 mmol/L (women)	<1.03 mmol/L

BMI body mass index; HDL-C high-density lipoprotein-cholesterol; HOMA-IR homeostasis model assessment; LDL-C low-density lipoprotein-cholesterol; WC waist circumference, TG triglycerides.

Supplementary Tables

Supplementary Table S2: Biomarkers of endotoxemia and type of dyslipidemia in survivors of childhood acute lymphoblastic leukemia: crude and adjusted models.

Biomarker	Low HDL	High LDL	High TG
	**OR (95 % CI)<br>*p* value**	**OR (95 % CI)<br>*p* value**	**OR (95 %CI) *p* value**
		**Crude models**	
LPS	0.67 (0.30 – 1.52)<br>0.337	1.12 (0.48 – 2.61)<br>0.796	0.59 (0.20 – 1.69)<br>0.321
LBP	**2.57 (1.38 – 4.82)<br>0.003**	1.86 (0.95 – 3.64)<br>0.071	1.57 (0.73 – 3.38)<br>0.254
		**Adjusted models**	
LPS	0.71 (0.31 – 1.63)<br>0.421	0.90 (0.35 – 2.36)<br>0.833	0.72 (0.25 – 2.10)<br>0.546
CRT	0.91 (0.35 – 2.33)<br>0.837	5.99 (1.39 – 25.79)<br>0.016	1.09 (0.31 – 3.88)<br>0.894
Age at diagnosis	1.03 (0.94 – 1.14)<br>0.502	0.93 (0.83 – 1.05)<br>0.238	1.10 (0.98 – 1.24)<br>0.100
Time since diagnosis	1.04 (0.96 – 1.12)<br>0.358	1.17 (1.06 – 1.30)<br>0.002	0.99 (0.90 – 1.09)<br>0.843
Gender	1.00 (0.43 – 2.30)<br>0.995	1.10 (0.41 – 2.92)<br>0.855	1.75 (0.61 – 5.08)<br>0.301
LBP	**2.39 (1.24 – 4.61)<br>0.010**	1.43 (0.67 – 3.04)<br>0.355	1.39 (0.62 – 3.13)<br>0.421
CRT	0.96 (0.49 – 1.90)<br>0.912	4.97 (1.87 – 13.23)<br>0.001	0.96 (0.41 – 2.26)<br>0.930
Age at diagnosis	1.01 (0.94 – 1.09)<br>0.744	0.97 (0.89 – 1.04)<br>0.366	1.06 (0.98 – 1.15)<br>0.165
Time since diagnosis	1.03 (0.97 – 1.10)<br>0.364	1.11 (1.03 – 1.20)<br>0.005	1.02 (0.95 – 1.10)<br>0.562
Gender	0.98 (0.52 – 1.86)<br>0.962	1.24 (0.59 – 2.61)<br>0.564	0.83 (0.36 – 1.83)<br>0.644

Supplementary Tables

Associations between biomarkers of endotoxemia and types of disturbances that define dyslipidemia: high triglycerides, high LDL-C, and low HDL-C in survivors of childhood acute lymphoblastic leukemia: crude and adjusted models. The crude and adjusted models were assessed between each biomarker and each type of dyslipidemia. Models were adjusted for CRT exposure, age at diagnosis, time since diagnosis and gender. Odds ratio (non-corrected 95% CI) and p-value are indicated for each association. Significant associations are in boldface. Bonferroni-adjusted alpha=0.05 / number of biomarkers = 0.05 / 2 = 0.025. CI confidence interval; CRT cranial radiotherapy; HDL-C high-density lipoprotein-cholesterol; LBP lipopolysaccharide-binding protein; LDL-C low-density lipoprotein-cholesterol; LPS lipopolysaccharide, TG triglycerides.

Supplementary Tables

Supplementary Table S3: Biomarkers of inflammation and type of dyslipidemia in survivors of childhood acute lymphoblastic leukemia: crude and adjusted models.

**Biomarker**	**Low HDL**<br>**OR (95 % CI)**<br>***p* value**	**High LDL**<br>**OR (95 % CI)**<br>***p* value**	**High TG**<br>**OR (95 % CI)**<br>***p* value**
		**Crude models**	
Adiponectin	0.42 (0.21 – 0.83)<br>0.013	0.71 (0.34 – 1.48)<br>0.355	0.40 (0.16 – 1.02)<br>0.055
Leptin	1.68 (0.83 – 3.39)<br>0.149	0.61 (0.29 – 1.31)<br>0.204	2.17 (0.84 – 5.62)<br>0.109
Ratio Leptin:Adiponectin	**2.84 (1.37 – 5.91)**<br>**0.005**	1.09 (0.52 – 2.31)<br>0.817	2.76 (1.03 – 7.37)<br>0.043
Resistin	1.14 (0.48 – 2.74)<br>0.767	0.88 (0.30 – 2.64)<br>0.823	3.72 (1.01 – 13.74)<br>0.050
Visfatin	0.80 (0.40 – 1.59)<br>0.517	0.71 (0.34 – 1.50)<br>0.371	0.83 (0.34 – 2.02)<br>0.678
IL-6	1.39 (0.77 – 2.53)<br>0.278	0.95 (0.49 – 1.86)<br>0.889	1.48 (0.68 – 3.23)<br>0.321
TNF-α	1.07 (0.59 – 1.93)<br>0.826	1.08 (0.56 – 2.08)<br>0.822	1.37 (0.64 – 2.94)<br>0.417
PAI-1	1.87 (0.90 – 3.90)<br>0.094	1.60 (0.74 – 3.47)<br>0.232	2.57 (0.95 – 6.97)<br>0.063
CRP	**2.58 (1.38 – 4.83)**<br>**0.003**	1.93 (0.97 – 3.84)<br>0.063	2.61 (1.17 – 5.84)<br>0.020
		**Adjusted models**	
Adiponectin	0.37 (0.18 – 0.77)<br>0.007	0.79 (0.34 – 1.81)<br>0.577	0.39 (0.15 – 0.98)<br>0.046
CRT	0.86 (0.40 – 1.86)<br>0.697	6.27 (1.88 – 20.86)<br>0.003	0.69 (0.26 – 1.82)<br>0.449
Age at diagnosis	1.04 (0.96 – 1.13)<br>0.305	0.94 (0.86 – 1.04)<br>0.212	1.08 (0.98 – 1.19)<br>0.136
Time since diagnosis	1.06 (0.99 – 1.13)<br>0.092	1.15 (1.06 – 1.25)<br>0.001	1.03 (0.95 – 1.12)<br>0.509

Supplementary Tables

Gender	0.67 (0.32 – 1.38)<br>0.277	1.23 (0.53 – 2.85)<br>0.629	0.93 (0.38 – 2.31)<br>0.883
Leptin	1.98 (0.80 – 4.90)<br>0.139	0.42 (0.14 – 1.25)<br>0.118	3.39 (1.03 – 11.14)<br>0.044
CRT	0.79 (0.35 – 1.80)<br>0.580	6.20 (1.75 – 22.01)<br>0.005	0.51 (0.18 – 1.49)<br>0.219
Age at diagnosis	1.06 (0.98 – 1.16)<br>0.165	0.95 (0.86 – 1.05)<br>0.327	1.11 (1.00 – 1.23)<br>0.055
Time since diagnosis	1.06 (0.99 – 1.13)<br>0.095	1.16 (1.07 – 1.27)<br>0.001	1.03 (0.95 – 1.12)<br>0.509
Gender	1.54 (0.61 – 3.88)<br>0.356	0.77 (0.26 – 2.30)<br>0.640	2.53 (0.78 – 8.21)<br>0.123
Ratio Leptin:Adiponectin	3.05 (1.36 – 6.81)<br>0.007	1.25 (0.52 – 3.05)<br>0.617	3.18 (1.10 – 9.18)<br>0.032
CRT	0.84 (0.36 – 1.93)<br>0.676	5.26 (1.54 – 17.94)<br>0.008	0.54 (0.19 – 1.57)<br>0.260
Age at diagnosis	1.06 (0.97 – 1.16)<br>0.193	0.95 (0.86 – 1.05)<br>0.332	1.11 (1.00 – 1.24)<br>0.050
Time since diagnosis	1.05 (0.99 – 1.13)<br>0.120	1.15 (1.06 – 1.25)<br>0.001	1.02 (0.94 – 1.11)<br>0.567
Gender	1.55 (0.69 – 3.47)<br>0.291	1.46 (0.59 – 3.60)<br>0.411	1.91 (0.68 – 5.32)<br>0.219
Resistin	1.14 (0.45 – 2.93)<br>0.783	0.73 (0.22 – 2.42)<br>0.604	4.33 (1.04 – 18.00)<br>0.044
CRT	0.68 (0.24 – 1.90)<br>0.461	3.47 (0.78 – 15.43)<br>0.102	0.20 (0.04 – 0.94)<br>0.041
Age at diagnosis	1.06 (0.95 – 1.18)<br>0.286	0.91 (0.79 – 1.04)<br>0.172	1.23 (1.05 – 1.44)<br>0.009
Time since diagnosis	1.11 (1.02 – 1.20)<br>0.013	1.12 (1.02 – 1.24)<br>0.024	1.06 (0.95 – 1.18)<br>0.300
Gender	1.61 (0.62 – 4.15)<br>0.325	0.80 (0.25 – 2.61)<br>0.711	0.98 (0.26 – 3.64)<br>0.976
Visfatin	0.83 (0.40 – 1.71)<br>0.615	0.77 (0.33 – 1.78)<br>0.539	0.75 (0.30 – 1.89)<br>0.538

Supplementary Tables

CRT	0.85 (0.38 – 1.91)<br>0.694	5.46 (1.60 – 18.65)<br>0.007	0.47 (0.17 – 1.31)<br>0.147
Age at diagnosis	1.06 (0.97 – 1.15)<br>0.197	0.95 (0.86 – 1.05)<br>0.290	1.11 (1.00 – 1.23)<br>0.051
Time since diagnosis	1.06 (0.99 – 1.13)<br>0.091	1.15 (1.06 – 1.25)<br>0.001	1.03 (0.94 – 1.12)<br>0.467
Gender	1.04 (0.50 – 2.15)<br>0.920	1.37 (0.59 – 3.20)<br>0.467	1.47 (0.58 – 3.73)<br>0.413
IL-6	1.37 (0.75 – 2.50)<br>0.307	0.96 (0.47 – 1.95)<br>0.902	1.38 (0.64 – 2.99)<br>0.412
CRT	1.06 (0.55 – 2.07)<br>0.862	4.78 (1.80 – 12.72)<br>0.002	0.94 (0.40 – 2.20)<br>0.879
Age at diagnosis	1.02 (0.95 – 1.09)<br>0.623	0.97 (0.90 – 1.05)<br>0.447	1.06 (0.98 – 1.15)<br>0.158
Time since diagnosis	1.05 (0.99 – 1.11)<br>0.130	1.12 (1.04 – 1.20)<br>0.003	1.02 (0.95 – 1.10)<br>0.605
Gender	0.78 (0.42 – 1.42)<br>0.407	1.08 (0.53 – 2.20)<br>0.837	0.72 (0.33 – 1.55)<br>0.400
TNF-α	1.17 (0.64 – 2.14)<br>0.607	1.21 (0.59 – 2.45)<br>0.605	1.48 (0.69 – 3.18)<br>0.314
CRT	1.08 (0.55 – 2.10)<br>0.825	5.25 (1.98 – 13.92)<br>0.001	1.00 (0.43 – 2.34)<br>0.995
Age at diagnosis	1.02 (0.95 – 1.09)<br>0.576	0.96 (0.89 – 1.04)<br>0.326	1.06 (0.98 – 1.15)<br>0.152
Time since diagnosis	1.05 (0.99 – 1.12)<br>0.117	1.12 (1.04 – 1.20)<br>0.003	1.03 (0.95 – 1.11)<br>0.459
Gender	0.74 (0.40 – 1.35)<br>0.326	1.13 (0.56 – 2.30)<br>0.737	0.72 (0.33 – 1.54)<br>0.396
PAI-1	1.79 (0.84 – 3.79)<br>0.131	1.34 (0.56 – 3.21)<br>0.509	2.80 (1.00 – 7.80)<br>0.050
CRT	0.65 (0.28 – 1.55)<br>0.331	5.19 (1.49 – 18.00)<br>0.001	0.41 (0.13 – 1.28)<br>0.126
Age at diagnosis	1.05 (0.96 – 1.14)<br>0.307	0.93 (0.84 – 1.03)<br>0.159	1.10 (0.99 – 1.22)<br>0.074

Supplementary Tables

Time since diagnosis	1.05 (0.98 – 1.12)<br>0.201	1.17 (1.07 – 1.27)<br>0.001	1.00 (0.91 – 1.09)<br>0.944
Gender	1.02 (0.49 – 2.15)<br>0.954	1.26 (0.53 – 2.99)<br>0.601	1.26 (0.49 – 3.27)<br>0.629
CRP	2.41 (1.24 – 4.67)<br>0.009	1.99 (0.91 – 4.35)<br>0.090	2.26 (0.98 – 5.21)<br>0.057
CRT	0.97 (0.49 – 1.90)<br>0.923	4.98 (1.87 – 13.26)<br>0.001	0.88 (0.37 – 2.08)<br>0.765
Age at diagnosis	1.02 (0.95 – 1.09)<br>0.638	0.96 (0.89 – 1.04)<br>0.357	1.04 (0.95 – 1.13)<br>0.441
Time since diagnosis	1.05 (0.98 – 1.11)<br>0.164	1.11 (1.03 – 1.19)<br>0.006	1.01 (0.93 – 1.09)<br>0.895
Gender	1.01 (0.53 – 1.93)<br>0.971	1.39 (0.65 – 2.98)<br>0.396	0.73 (0.31 – 1.69)<br>0.460

Associations between biomarkers of inflammation and types of disturbances that define dyslipidemia: high triglycerides, high LDL-C, and low HDL-C levels in survivors of childhood acute lymphoblastic leukemia: crude and adjusted models. The crude and adjusted models were assessed between each biomarker and each type of dyslipidemia. Models were adjusted for CRT exposure, age at diagnosis, time since diagnosis and gender. Odds ratio (non-corrected 95% CI) and p-value are indicated for each association. Significant associations are in boldface. Bonferroni-adjusted alpha=0.05 / number of biomarkers = 0.05 / 9 = 0.006. CI confidence interval; CRP C-reactive protein; CRT cranial radiotherapy; HDL-C high-density lipoprotein-cholesterol; IL-6 interleukin-6; LDL-C low-density lipoprotein-cholesterol; PAI-1 plasminogen activator inhibitor-1; TNF-α tumor necrosis factor-α; TG triglycerides.

Supplementary Tables

Supplementary Table S4: Biomarkers of oxidative stress and type of dyslipidemia in survivors of childhood acute lymphoblastic leukemia: crude and adjusted models.

Biomarker	Low HDL<br>OR (95 % CI)<br>*p* value	High LDL<br>OR (95 % CI)<br>*p* value	High TG<br>OR (95 % CI)<br>*p* value
		**Crude models**	
GSH	1.26 (0.69 – 2.30)<br>0.448	0.59 (0.30 – 1.17)<br>0.131	1.26 (0.58 – 2.72)<br>0.558
GPx	1.37 (0.76 – 2.49)<br>0.296	1.25 (0.65 – 2.43)<br>0.504	1.83 (0.84 – 3.98)<br>0.130
Protein carbonyls	1.14 (0.48 – 2.74)<br>0.767	0.88 (0.30 – 2.64)<br>0.823	0.61 (0.19 – 2.00)<br>0.416
SOD	1.00 (0.50 – 2.00)<br>1.000	1.16 (0.54 – 2.47)<br>0.704	0.73 (0.29– 1.81)<br>0.495
mtDNA	1.23 (0.66 – 2.29)<br>0.510	0.63 (0.31 – 1.31)<br>0.219	2.43 (1.03 – 5.75)<br>0.044
		**Adjusted models**	
GSH	1.31 (0.71 – 2.41)<br>0.392	0.75 (0.36 – 1.56)<br>0.444	1.24 (0.57 – 2.68)<br>0.589
CRT	1.16 (0.59 – 2.30)<br>0.663	4.77 (1.79 – 12.77)<br>0.002	0.97 (0.41 – 2.31)<br>0.941
Age at diagnosis	1.02 (0.95 – 1.09)<br>0.677	0.97 (0.89 – 1.04)<br>0.373	1.06 (0.98 – 1.15)<br>0.157
Time since diagnosis	1.05 (0.99 – 1.12)<br>0.115	1.12 (1.04 – 1.20)<br>0.004	1.03 (0.95 – 1.11)<br>0.495
Gender	0.86 (0.46 – 1.58)<br>0.621	1.16 (0.57 – 2.40)<br>0.680	0.85 (0.39 – 1.83)<br>0.669
GPx	1.43 (0.78 – 2.61)<br>0.243	1.37 (0.67 – 2.78)<br>0.385	1.87 (0.86 – 4.07)<br>0.110
CRT	1.07 (0.55 – 2.09)<br>0.843	5.05 (1.90 – 13.44)<br>0.001	0.96 (0.41 – 2.25)<br>0.840
Age at diagnosis	1.02 (0.95 – 1.09)<br>0.597	0.97 (0.89 – 1.04)<br>0.373	1.06 (0.98 – 1.15)<br>0.132

Supplementary Tables

Time since diagnosis	1.05 (0.99 – 1.12)<br>0.106	1.12 (1.04 – 1.20)<br>0.003	1.03 (0.96 – 1.11)<br>0.378
Gender	0.76 (0.41 – 1.38)<br>0.364	1.11 (0.54 – 2.25)<br>0.783	0.76 (0.36 – 1.63)<br>0.528
Protein carbonyls	1.10 (0.42 – 2.84)<br>0.849	0.78 (0.23 – 2.71)<br>0.701	0.42 (0.11 – 1.53)<br>0.187
CRT	0.67 (0.24 – 1.88)<br>0.448	3.60 (0.81 – 15.97)<br>0.092	0.19 (0.04 – 0.85)<br>0.030
Age at diagnosis	1.06 (0.95 – 1.17)<br>0.312	0.92 (0.80 – 1.05)<br>0.207	1.21 (1.04 – 1.41)<br>0.013
Time since diagnosis	1.11 (1.02 – 1.20)<br>0.013	1.12 (1.02 – 1.24)<br>0.025	1.06 (0.96 – 1.17)<br>0.288
Gender	1.61 (0.61 – 4.22)<br>0.333	0.74 (0.21 – 2.56)<br>0.631	0.53 (0.15 – 1.91)<br>0.333
SOD	0.97 (0.46 – 2.02)<br>0.925	1.07 (0.45 – 2.52)<br>0.878	0.81 (0.31 – 2.12)<br>0.666
CRT	0.86 (0.38 – 1.94)<br>0.713	5.18 (1.52 – 17.70)<br>0.009	0.56 (0.20 – 1.60)<br>0.276
Age at diagnosis	1.06 (0.98 – 1.16)<br>0.170	0.96 (0.87 – 1.06)<br>0.392	1.11 (1.00 – 1.23)<br>0.053
Time since diagnosis	1.06 (0.99 – 1.13)<br>0.073	1.15 (1.06 – 1.25)<br>0.001	1.03 (0.95 – 1.12)<br>0.446
Gender	1.03 (0.49 – 2.15)<br>0.948	1.30 (0.55 – 3.10)<br>0.552	1.17 (0.45 – 3.04)<br>0.746
mtDNA	1.33 (0.71 – 2.48)<br>0.374	0.74 (0.34 – 1.59)<br>0.439	2.54 (1.08 – 6.01)<br>0.034
CRT	1.06 (0.53 – 2.10)<br>0.876	5.00 (1.73 – 14.48)<br>0.003	0.93 (0.38 – 2.29)<br>0.872
Age at diagnosis	1.01 (0.95 – 1.09)<br>0.692	0.97 (0.90 – 1.06)<br>0.531	1.08 (0.99 – 1.18)<br>0.076
Time since diagnosis	1.05 (0.99 – 1.11)<br>0.135	1.09 (1.01 – 1.18)<br>0.021	1.03 (0.96 – 1.12)<br>0.408
Gender	0.80 (0.43 – 1.51)<br>0.491	1.31 (0.61 – 2.82)<br>0.489	0.64 (0.28 – 1.46)<br>0.288

Supplementary Tables

Associations between biomarkers of oxidative stress and types of disturbances that define dyslipidemia: high triglycerides, high LDL-C, and low HDL-C in survivors of childhood acute lymphoblastic leukemia: crude and adjusted models. The crude and adjusted models were assessed between each biomarker and each type of dyslipidemia. Models were adjusted for CRT exposure, age at diagnosis, time since diagnosis and gender. Odds ratio (non-corrected 95% CI) and p-value are indicated for each association. Bonferroni-adjusted alpha=0.05 / number of biomarkers = 0.05 / 5 = 0.01. CI confidence interval; CRT cranial radiotherapy; GPx glutathione peroxidase; GSH glutathione; HDL-C high-density lipoprotein-cholesterol; LDL-C low-density lipoprotein-cholesterol; mtDNA mitochondrial DNA; SOD superoxide dismutase; TG triglycerides.

Supplementary Tables

Supplementary Table S5: Biomarkers of endothelial function and type of dyslipidemia in survivors of childhood acute lymphoblastic leukemia: crude and adjusted models.

Biomarker	Low HDL<br>OR (95 % CI)<br>*p* value	High LDL<br>OR (95 % CI)<br>*p* value	High TG<br>OR (95 % CI)<br>*p* value
		**Crude models**	
ICAM -1	1.51 (0.83 – 2.73)<br>0.180	1.32 (068 – 2.55)<br>0.408	1.83 (0.84 – 3.98)<br>0.130
VCAM -1	0.73 (0.40 – 1.32)<br>0.296	0.54 (0.28 – 1.05)<br>0.071	0.55 (0.25 – 1.19)<br>0.130
E -selectin	1.77 (0.70 – 4.47)<br>0.224	2.89 (0.85 – 9.79)<br>0.090	1.03 (0.31 – 3.42)<br>0.962
		**Adjusted models**	
ICAM -1	1.43 (0.78 – 2.63)<br>0.248	1.17 (0.58 – 2.38)<br>0.667	1.91 (0.87 – 4.20)<br>0.109
CRT	1.11 (0.57 – 2.16)<br>0.769	5.31 (2.00 – 14.12)<br>0.001	1.04 (0.44 – 2.47)<br>0.925
Age at diagnosis	1.02 (0.96 – 1.09)<br>0.533	0.96 (0.89 – 1.04)<br>0.330	1.07 (0.98 – 1.16)<br>0.126
Time since diagnosis	1.04 (0.98 – 1.11)<br>0.178	1.11 (1.03 – 1.19)<br>0.005	1.02 (0.94 – 1.10)<br>0.691
Gender	0.76 (0.42 – 1.40)<br>0.381	1.16 (0.57 – 2.34)<br>0.685	0.78 (0.36 – 1.67)<br>0.519
VCAM -1	0.92 (0.47 – 1.77)<br>0.791	0.71 (0.33 – 1.54)<br>0.384	0.64 (0.27 – 1.49)<br>0.310
CRT	1.08 (0.55 – 2.09)<br>0.828	5.21 (1.97 – 13.80)<br>0.001	0.99 (0.43 – 2.30)<br>0.982
Age at diagnosis	1.02 (0.95 – 1.09)<br>0.641	0.96 (0.88 – 1.03)<br>0.258	1.05 (0.97 – 1.14)<br>0.264
Time since diagnosis	1.04 (0.98 – 1.11)<br>0.169	1.10 (1.02 – 1.19)<br>0.012	1.01 (0.94 – 1.09)<br>0.747

Supplementary Tables

Gender	0.77 (0.41 – 1.44)<br>0.414	1.26 (0.61 – 2.61)<br>0.534	0.85 (0.39 – 1.88)<br>0.692
E -selectin	1.74 (0.65 – 4.66)<br>0.268	4.56 (1.14 – 18.17)<br>0.032	1.13 (0.32 – 4.02)<br>0.855
CRT	0.80 (0.26 – 2.42)<br>0.692	3.93 (0.75 – 20.55)<br>0.106	0.19 (0.04 – 0.90)<br>0.037
Age at diagnosis	1.05 (0.95 – 1.17)<br>0.331	0.89 (0.77 – 1.04)<br>0.143	1.20 (1.03 – 1.39)<br>0.019
Time since diagnosis	1.10 (1.01 – 1.19)<br>0.023	1.13 (1.02 – 1.26)<br>0.022	1.04 (0.94 – 1.15)<br>0.425
Gender	1.47 (0.54 – 3.98)<br>0.451	0.40 (0.10 – 1.57)<br>0.190	0.61 (0.17 – 2.19)<br>0.444

Associations between biomarkers of endothelial function and types of disturbances that define dyslipidemia: high triglycerides, high LDL-C, and low HDL-C in survivors of childhood acute lymphoblastic leukemia: crude and adjusted models. The crude and adjusted models were assessed between each biomarker and each type of dyslipidemia. Models were adjusted for CRT exposure, age at diagnosis, time since diagnosis and gender. Odds ratio (non-corrected 95% CI) and p-value are indicated for each association. Bonferroni-adjusted alpha=0.05 / number of biomarkers = 0.05 / 3 = 0.017. CI confidence interval; CRT cranial radiotherapy; HDL-C high-density lipoprotein-cholesterol; ICAM-1 intercellular adhesion molecule-1; LDL-C low-density lipoprotein-cholesterol; TG triglycerides; VCAM-1 vascular cell adhesion molecule-1.
